# Aromatase Derived Estradiol Within the Thalamus Modulates Pain Induced by Varicella Zoster Virus

**DOI:** 10.3389/fnint.2018.00046

**Published:** 2018-10-12

**Authors:** Phillip R. Kramer, Mahesh Rao, Crystal Stinson, Larry L. Bellinger, Paul R. Kinchington, Michael B. Yee

**Affiliations:** ^1^Department of Biomedical Science, Texas A&M University College of Dentistry, Dallas, TX, United States; ^2^Department of Ophthalmology and of Molecular Microbiology and Genetics, Eye and Ear Foundation, School of Medicine, University of Pittsburgh, Pittsburgh, PA, United States

**Keywords:** orofacial, herpes zostr, neuralgia, shingles, pain, estrogen, aromatase, thalamus

## Abstract

Herpes zoster or shingles is the result of varicella zoster virus (VZV) infection and often results in chronic pain that lasts for months after visible symptoms subside. Testosterone often attenuates pain in males. Previous work demonstrates ovarian estrogen effects γ-aminobutyric acid (GABA) signaling in the thalamus, reducing pain but the role of testosterone within the thalamus is currently unknown. Because aromatase affects pain and is present in the thalamus we tested a hypothesis that testosterone converted to estrogen in the thalamus attenuates herpes zoster induced pain. To address this hypothesis, male Sprague-Dawley rats received whisker pad injection of either MeWo cells or MeWo cells containing VZV. To reduce aromatase derived estrogen in these animals we injected aromatase inhibitor letrozole systemically or infused it into the thalamus. To test if estrogen was working through the estrogen receptor (ER) agonist, 4, 4′, 4″-(4-Propyl-[1*H*]-pyrazole-1,3,5-triyl)*tris*phenol (PPT) was infused concomitant with letrozole. Motivational and affective pain was measured after letrozole and/or PPT treatment. Vesicular GABA transporter (VGAT) is important in pain signaling. Because estrogen effects VGAT expression we measured its transcript and protein levels after letrozole treatment. Virus injection and letrozole significantly increased the pain response but thalamic infusion of PPT reduced zoster pain. Letrozole increased the number of thalamic neurons staining for phosphorylated ERK (pERK) but decreased VGAT expression. The results suggest in male rats aromatase derived estradiol interacts with the ER to increase VGAT expression and increase neuronal inhibition in the thalamus to attenuate VZV induced pain.

## Introduction

Herpes zoster is caused by infection of the varicella zoster virus (VZV). The virus results in chicken pox and remains latent in sensory nerve ganglia (Mahalingam et al., 1992). The virus reactivates and triggers herpes zoster, also known as shingles (Donahue et al., 1995; Jung et al., 2004). The clinical presentation of VZV infection usually involves neuronal inflammation, blisters and a dermatomal rash causing severe itching (Cohen, 2013). This outbreak of shingles is associated with pain during the early phase that then abates. Pain can return after a few months (>3 months), potentially due to inflammation and nerve damage (Gershon et al., 2015) along one or more sensory nerve roots (Soares and Provenzale, 2016).

Almost a million incidences of herpes zoster are reported annually in the US (Donahue et al., 1995; Johnson et al., 2015). The Advisory Committee on Immunization Practices mentioned in their report that one in every three persons develops herpes zoster during their life-time (Harpaz et al., 2008). In spite of immunity due to vaccination, about 20% of patients develop chronic pain (Harpaz et al., 2008). Although herpes zoster incidence increases after 50 years of age, it can affect all ages (Civen et al., 2009). Unfortunately, herpes zoster pain severely effects the quality of life for many patients suffering from this disease (Johnson et al., 2010).

Modulation of pain sensitivity by steroids, especially by estradiol, has been widely studied (Craft et al., 2004; Craft, 2007; Aloisi and Sorda, 2011; Palmeira et al., 2011; Amandusson and Blomqvist, 2013; Pieretti et al., 2016). Sex steroids like estrogen can increase activity in the thalamus of stressed rats (Ueyama et al., 2006). Our lab has demonstrated that proestrus estradiol alters γ-aminobutyric acid (GABA)ergic gene expression, for example increasing vesicular GABA transporter (VGAT), in the thalamic region resulting in altered pain responses (Umorin et al., 2016). Sex steroids influence the excitability of neurons by altering GABA amino decarboxylase (GAD) and GABA_A_ receptor subunits expression (Jüptner et al., 1991; Noriega et al., 2010). Sex steroids can also act as a neurotransmitter, for example progesterone metabolites can bind the GABA receptor potentiating neuronal inhibition (Majewska et al., 1986). Testosterone reduces orofacial pain (Fischer et al., 2007) moreover, male rats show a reduced response to VZV induced pain (Stinson et al., 2017). Thus, sex steroids have a proven role in altering neurotransmission and show evidence of effecting pain that could involve genomic and non-genomic mechanisms within the thalamus.

Patients undergoing aromatase inhibitor therapy often discontinue their treatment due to pain in their joints and muscles (Henry et al., 2008, 2012). In animals pain increased after administering aromatase inhibitors; potentially through increased neuronal excitability (Robarge et al., 2016). Moreover, blockade of aromatase increases the pain responses in rats (Ghorbanpoor et al., 2014; Robarge et al., 2016). These results suggest estrogen derived from aromatase is important in controlling pain.

Previous work in our lab demonstrated that sex steroids and thalamic GABAergic signaling could have a role in controlling orofacial pain (Umorin et al., 2016; Stinson et al., 2017) but the role of testosterone within the thalamus has not been studied. Aromatase converts testosterone to estradiol in the thalamus (Biegon et al., 2010). Thus, it was hypothesized that thalamic testosterone once converted to estrogen attenuates herpes zoster induced pain. To address this question our lab modulated the level of aromatase in the thalamic region and then measured the pain response after VZV injection. Further, VGAT was quantitated within the thalamus to characterize the role of inhibitory neurons within the reticular thalamic nuclei (Rt). Thalamic infusion of an estrogen receptor (ER) agonist was completed to identify if aromatase derived estradiol was inducing its action on behavior through the ER.

## Materials and Methods

### Animal Husbandry

This study was carried out in accordance with the recommendations of Institutional Animal Care and Use Committee Guidebook, Texas A&M University College of Dentistry Institutional Animal Care and Use Committee. The protocol was approved by the Texas A&M University College of Dentistry Institutional Animal Care and Use Committee. Adult male Sprague-Dawley rats (300 g) from Envigo (Huntingdon, UK) were kept on a 12:12 light/dark cycle. The rats were given food and water *ad libitum*.

### Treatment and Experimental Groups

Table 1 summarizes the four experiments and each treatment group. Rats in the first experiment were divided so that half received a whisker pad injection of 100 μl of MeWo cells infected with VZV (>50,000–60,000 pfu/μl) or the same volume of control MeWo cells (human skin cell line) lacking virus. Starting 1 week after whisker pad injection the animals received a 120 μl subcutaneous injection of 5 mg/ml letrozole or vehicle each week 1 h before behavioral testing. Dimethyl sulfoxide (DMSO; 120 μl) was injected as a vehicle. Testing 1 to 3 h after administering drug produced the maximal behavioral response in preliminary studies. The groups were control/vehicle, control/letrozole, VZV/vehicle, VZV/letrozole (six animals per group).

**Table 1:** Experimental outline for testing varicella zoster virus (VZV) pain.

Experiment	Question	Experimental groups		Experimental tests		(Number of animals)
Experiment #1: systemic injection of 5 mg/ml letrozole after whisker pad injection with VZV	Can inhibition of aromatase alter VZV induced pain?	Whisker pad injection	Sub-Q injection			
		Control	Vehicle	Affective pain		(6)
		Control	Letrozole 5 mg/ml	Affective pain		(6)
		VZV	Vehicle	Affective pain		(6)
		VZV	Letrozole 5 mg/ml	Affective pain		(6)
Experiment #2: thalamic infusion of 5 mg/ml letrozole after whisker pad injection with VZV	Can inhibition of aromatase specifically within the thalamus alter VZV induced pain?	Whisker pad injection	Thalamic infusion				
		Control	Vehicle	Affective pain		(9)
		Control	Letrozole 5 mg/ml	Affective pain		(9)
		VZV	Vehicle	Affective pain		(9)
		VZV	Letrozole 5 mg/ml	Affective pain		(9)
		Control	Vehicle	RT-PCR (4)	ICC (5)	
		Control	Letrozole 5 mg/ml	RT-PCR (4)	ICC (5)	
		VZV	Vehicle	RT-PCR (4)	ICC (5)	
		VZV	Letrozole 5 mg/ml	RT-PCR (4)	ICC (5)	
Experiment #3: thalamic infusion of 1 mg/ml and 5 mg/ml letrozole and whisker pad injection with VZV	Does the dosage of aromatase inhibitor infused into the thalamus effect the VZV induced pain?	Whisker pad injection	Thalamic infusion			
		Control	Vehicle	Affective pain ELISA		(5)
		Control	Letrozole 5 mg/ml	Affective pain ELISA		(5)
		VZV	Vehicle	Affective pain ELISA		(5)
		VZV	Letrozole 1 mg/ml	Affective pain ELISA		(5)
		VZV	Letrozole 5 mg/ml	Affective pain ELISA		(5)
Experiment #4: thalamic infusion 5 mg/ml letrozole and estrogen receptor agonist PPT after whisker pad injection with VZV	Does the estrogen produced by aromatase in the thalamus alter VZV induced pain by binding to the estrogen receptor	Whisker pad injection	Thalamic infusion		
		Control	PPT	Affective pain		(8)
		Control	PPT and letrozole 5 mg/ml	Affective pain		(8)
		VZV	PPT	Affective pain		(8)
		VZV	PPT and letrozole 5 mg/ml	Affective pain		(8)
		Control	PPT	ICC		(4)
		Control	PPT and letrozole 5 mg/ml	ICC		(4)
		VZV	PPT	ICC		(4)
		VZV	PPT and letrozole 5 mg/ml	ICC		(4)

*Abbreviations: VZV, varicella zoster virus; ICC, immunocytochemistry; RT-PCR, real time polymerase chain reaction; PPT, 4, 4′, 4″-(4-Propyl-[1*H*]-pyrazole-1,3,5-triyl)*tris*phenol; ELISA, Enzyme-linked immunosorbent assay*.

Previous studies suggested a minimal dose of 2 mg/kg of letrozole will block signaling of the ER (Liu et al., 2015). In these studies rats weighing 300 g were injected with 120 μl of 5 mg/ml letrozole, equivalent to a 2 mg/kg dose. Letrozole is permeable to the blood brain barrier (Dave et al., 2013).

Rats in the second experiment received a whisker pad injection of VZV or control. One week after whisker pad injection these animals were divided so that they received thalamic infusion of letrozole or vehicle each week, 1 h before behavioral testing (nine animals per group). Of these nine animals four were used for real-time PCR (RT-PCR) and five for immunocytochemistry (ICC). Rats in the third experiment were injected with VZV or control and a week later received thalamic infusion of different doses of letrozole each week, 1 h before testing (five animals per group). In a fourth experiment the rats were injected with VZV or control and after 1 week the thalamus was infused with aromatase inhibitor letrozole and/or ER agonist (4, 4^′^, 4^′′^-(4-Propyl-[1*H*]-pyrazole-1,3,5-triyl)*tris*phenol, PPT) each week before testing. The groups were control/PPT; control/PPT and letrozole; VZV/PPT; and VZV/PPT and letrozole with eight animals per group. ICC was completed on 8 of the animals.

### Thalamic Guide Cannula Surgery

In Experiments #2, 3 and 4 rats (300 g) underwent surgery to place a bilateral guide cannula into the thalamus 1 week after arrival to the vivarium. Rats were anesthetized with 2% isoflurane with an air flow of 2 liters per minute. Using sterile technique guide cannulas (C313G Plastics One, Roanoke, VA, USA) were placed bilaterally into the thalamus stereotaxically using the coordinates 3.6 mm posterior of Bregma, 3.0 mm from midline at a depth of 5.5 mm from the top of the skull. The guide cannulas were closed with obturators. Post-surgery the animals received nalbuphine (2 mg/kg) subcutaneously. This dose of analgesic given again after 24 h and the animals recovered for a week before whisker pad injection.

### Thalamic Infusion Protocol

For thalamic infusion the obturators were removed and an injection syringe (that projected 0.5 mm below the guide cannula) was inserted. Bilateral infusions (50 nanoliters/min) included letrozole (500 nanoliters at a concentration of 1 mg/ml or 5 mg/ml) and/or PPT (1 μM) or vehicle DMSO (500 nanoliters; Sun et al., 2002; Dave et al., 2013). After infusion, the injector cannula was left in place for 5 min, removed and the obturator replaced. Previous reports demonstrate that 500 nanoliters injected at these coordinates spreads to the posterior thalamic nucleus (Po), ventral posteromedial (VPM) and ventral posterolateral thalamic nuclei (VPL) and the Rt (Kramer et al., 2017).

### Behavioral Testing

Place Escape/Avoidance Paradigm (PEAP) testing was performed to determine pain. To accomplish this, the rats were placed in a 30 cm × 30 cm × 30 cm acrylic box where half the box was covered in black cloth. This test chamber was modeled from the PEAP test performed by the Fuchs’s laboratory (LaBuda and Fuchs, 2000). This assay was used to measure the motivation/affective aspect of pain (LaBuda and Fuchs, 2000; Baastrup et al., 2011). The PEAP test is based on the assumption that if animals escape and/or avoid a noxious stimulus, then the stimulus is aversive to the animal. Rodents being nocturnal in nature preferred to stay on the dark side when placed into the test chamber that has a light and dark side. After placing the rat in the test chamber, the rat was immediately poked with a 60-g filament every 15 s on the injected side if the rat was on the dark side and on the non-injected side if it was on the light side. The target region for the poking was the area below the eye and caudal to the whisker pad. This region is innervated by the second branch of trigeminal ganglion (DaSilva and DosSantos, 2012), the nerve infected by VZV injection of the whisker pad. The time spent on the dark side of the box was recorded in 5-min bins and testing was performed for a total of 30 min. Testing was performed once a week. Testing was completed for 2 weeks. Thus, the theory behind the test is that if the rat is experiencing VZV induced pain when poked in the sensitive area it will not stay on it preferred dark side but will move to the non-preferred light side and stay there to avoid the pain poke.

### Thalamic Tissue Punches

Fresh thalamic tissue (5–10 mg) was collected 1 h post PEAP testing. Animals were euthanized by exposure to CO_2_ followed by decapitation. The brain was removed using a rongeur and placed on a brain slicer (Zivic, Pittsburgh, PA, USA). After cooling 2 mm thick sections were cut between Bregma −3 to −5. These sections were placed on glass slides and kept on dry ice. Lateral thalamic tissue was collected with punches 2 mm in diameter centered on the injection site, punches included the Po, VPM, VPL and Rt. Tissue was stored in liquid nitrogen until use.

### Real Time PCR

RNA was isolated from thalamic tissue punches obtained from four randomly selected animals per group in Experiment #2 (see Table 1). RNA extraction was performed using the RNA Lipid Tissue Kit from Qiagen (Valencia, CA, USA). The RNA concentration in the resulting sample was determined on a Nanodrop2000. A one-step reverse transcription polymerase chain reaction (PCR) reaction was performed on BioRAD C1000 Thermal Cycler using the SYBR-Green 1-Step RT-PCR kit and primers from Qiagen (Table 2). The thermal protocol was 30 min @ 50°C for the reverse transcription reaction, 15 min @ 95°C for DNA pol activation and 40× (15 s @ 94°C melting, 30 s @ 56°C annealing, 30 s @ 72°C extension). A melting curve was obtained thereafter for quality assurance. Sample amount was adjusted according to total RNA concentration to obtain 20 ng of total RNA per well in the final reaction mix. All reactions were run in triplicate. PCR runs that did not exhibit a proper amplification profile were discarded. For each sample, the threshold Ct value for glyceraldehyde-3-phosphate dehydrogenase (GAPDH) was subtracted from the Ct of value for VGAT or the genes in Table 2 to give a ΔCt. Values for decreased expression were calculate by using the formula (−1/ΔCt).

**Table 2:** PCR primer pairs for RT-PCR.

Gene	Qiagen catalog #	Qiagen ID
GAPDH	QT00199633	Gapd
GPR30	QT00376943	Gper1
Estrogen receptor alpha	QT00369740	ESrra
Estrogen receptor beta	QTOO190113	Esr2
Aromatase receptor	QT01812433	Ar
Aromatase enzyme	QT00186942	Cyp19a1
VGAT	QT00378413	Viaat

### Enzyme-Linked Immunosorbent Assay (ELISA)

Fresh thalamic tissue punches from all five animals in Experiment #3 (Table 1) was stored in liquid nitrogen until analysis. Tissue was placed in 250 μl of T-Per tissue protein extraction reagent containing Halt Protease Inhibitor and ground (Thermo Scientific, Rockford, IL, USA). Ground samples were frozen and thawed, followed by centrifugation and decanting of the supernatant. Quantitation of VGAT in the supernatant was completed on duplicate 100 μl samples of supernatant using an SLC32A1 (VGAT) enzyme-linked immunosorbent assay (ELISA) following the manufacturer’s directions (Cusabio, catalog #CSB-EL021578MO). Total protein was determined in each sample using a BCA protein assay (Thermo Scientific, Waltham, WA, USA). Values represent the pg of VGAT per μg of total protein.

### Immuno-Fluorescent Staining

In Experiment #2, five animals were randomly selected from the nine animals and in Experiment #4, four animals were randomly selected from the eight animals (Table 1). After anesthesia with 100 mg/kg ketamine and 10 mg/kg xylazine the animals were perfused with 9% sucrose followed by 4% paraformaldehyde. Fixed tissues were stored in 25% sucrose, frozen, cryo-sectioned and the 32 μm sections placed on Histobond slides (VWR international, Radnor, PA, USA). The tissue was then blocked with a phosphate buffered saline (PBS) solution containing 5% normal goat serum (Sigma-Aldrich, St. Louis, MO, USA) and 0.3% Triton-X 100 for 2 h at room temperature. The slides were then incubated in a primary antibody solution overnight at 4°C. The primary antibody consisted of a mixture of the mouse monoclonal neuronal nuclei (NeuN) antibody (Millipore, Billerica, MA, USA, MAB377) 1:150 dilution or GAD 67 antibody (Millipore clone 1G10.2, MAB5406) 1:500 dilution and rabbit phosphorylated ERK (pERK) antibody (phosphorylated extracellular signal-regulated kinase, Cell Signaling Technology, Boston, MA, USA #4695) at 1:150 dilution. The primary antibody was diluted with PBS, 5% BSA and 0.3% Triton X-100. After incubation in primary antibody the slides were then rinsed three times in PBS and Triton-X 100 for a total of 45 min and placed for 2 h in secondary antibody and PBS and 0.3% Triton X-100. Secondary antibodies (1:500 dilution) included a mixture of goat anti-mouse 568 or goat anti-rabbit 488 (Invitrogen, Carlsbad, CA, USA). After rinsing the slides three times in PBS for a total of 45 min, the slides were mounted with Fluoromount-G mounting medium containing Hoechst 33342 stain (Electron Microscopy Sciences, Hatfield, PA, USA). The fluorescent signal was imaged using a Nikon fluorescent microscope and NIS-Elements imaging software and a Photometrics CoolSnap K4 CCD camera (Roper Scientific, Inc., Duluth, GA, USA).

GAD 67 expression is primarily in the cell body while GAD 65 is localized to nerve terminals (Kaufman et al., 1991). This lead to ease of identification of GABA positive cells and accurate counting of these cells. Moreover, GAD 67 expression is constitutive whereas GAD 65 expression changes with cellular activity (Fenalti et al., 2007). Thus, GAD 67 was used as a marker for counting the GABA cells.

Cell counts were completed by a blinded reviewer. The injection site was the center point from which sections were selected. Every other section was selected for staining. Typically, three sections were counted for each animal. The slides were analyzed using ImageJ software, the average background for the slides within a treatment group was subtracted from the image and a fluorescent signal associated with a cell nucleus was counted as a positive cell. Counts were completed for the number of GAD 67, NeuN, GAD 67/pERK and/or NeuN/pERK stained cells within a 0.125 mm^2^ field adjacent to the injection site. On each section two randomly selected fields near the tip of the injection site was counted. Counts were completed within the lateral thalamic nuclei (VMP, VPL and Rt) and cell counts from the two fields on each section were then averaged. This average count for the three sections was averaged for each animal. Values were given as a mean and standard error of the mean (SEM) representing an average of the values for the animals in each treatment group.

### Statistics

Data was tested with the KS normality test (α = 0.05). PEAP data passed the normality test and was analyzed by two-way ANOVA. The independent variables were the time spent on the light or dark side of the box (collected in six time bins i.e., 5, 10, 15, 20, 25 and 30 min) and the dependent variable was treatment (i.e., virus and drug). PEAP data was analyzed with repeated measures because data was collected multiple times over a 30-min period from each rat. When a significant effect was observed Bonferroni *post hoc* tests were completed. PCR, ELISA and cell count data was analyzed with the non-parametric Mann-Whitney test (Prizm 5.04, GraphPad Software, La Jolla, CA, USA or Abstat, Anderson Bell Corp., Arvada, CO, USA).

## Results

### Enhanced Pain Response After Systemic Letrozole Treatment

**Figure 1:**
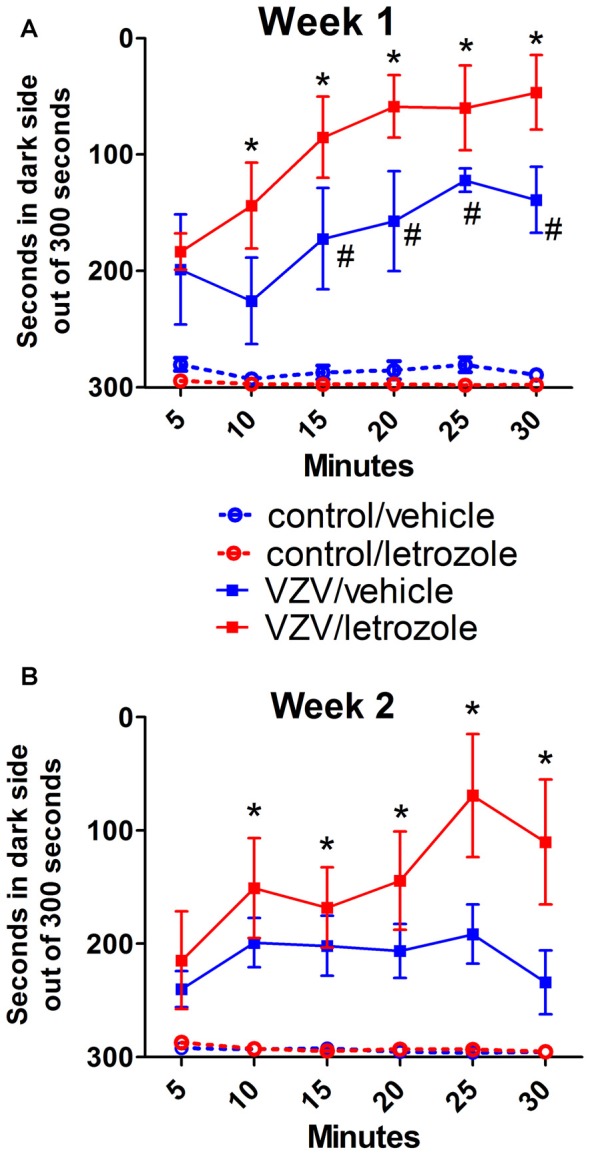
Systemic injection of aromatase inhibitor letrozole 5 mg/ml did not alter the varicella zoster virus (VZV) induced pain response. The hashtag symbol indicates a significant difference between the control/vehicle and VZV/vehicle groups (*p* < 0.05). The asterisks indicate a significant difference between the control/letrozole and VZV/letrozole groups (*p* < 0.05). Panel **(A)** is week 1 data and panel **(B)** is week 2 data. There were six animals per group. Values are means and SEM.

PEAP data from Experiment #1 (Figure 1) shows that in the first week the animals which received virus, whether given letrozole or vehicle spent much less time on the dark side compared to the controls *F*_(3,100)_ = 27.8, *p* < 0.0001. In week 2 there was also an effect of VZV treatment *F*_(3,100)_ = 9.8, *p* < 0.001. Animals in the control group remained on the dark side almost throughout the testing period. When comparing the virus group injected with vehicle to the virus group injected with letrozole no significant effect for letrozole was observed over the 2-week testing period (Figures 1A,B). A significant interaction between time and treatment was observed in the animals injected with letrozole in week 1 *F*_(15,100)_ = 2.6, *p* < 0.005 but not in week 2 *F*_(15,100)_ = 0.83, *p* = 0.63.

### Thalamic Infusion of Letrozole

**Figure 2:**
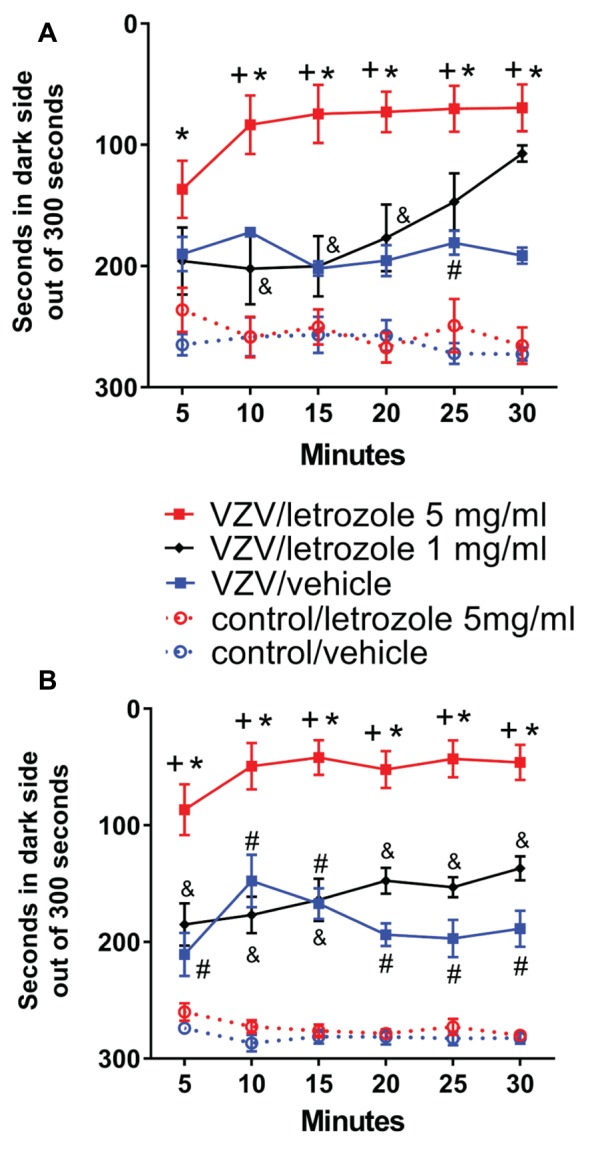
Local thalamic infusion of the aromatase inhibitor letrozole significantly increased the VZV induced pain response. The hashtag symbol indicates a significant difference between the control/vehicle and VZV/vehicle groups (*p* < 0.05). The asterisks indicate a significant difference between the control/letrozole 5 mg/ml and VZV/letrozole 5 mg/ml groups (*p* < 0.05). The plus sign indicates a significant difference between the VZV/vehicle and VZV/letrozole 5 mg/ml groups (*p* < 0.05). The ampersand symbol indicates a significant difference between the VZV/letrozole 1 mg/ml and the VZV/letrozole 5 mg/ml groups (*p* < 0.05). Panel **(A)** is week 1 data and panel **(B)** is week 2 data, *n* = 9 per group. Values are means and SEM.

Thalamic infusion of letrozole in Experiment #2 significantly increased the pain response in week 1 *F*_(3,125)_ = 34.9, *p* < 0.0001. In week 2 there was also an effect of letrozole treatment *F*_(3,125)_ = 109.2, *p* < 0.0001. VZV injection significantly increased the pain response in week 1 and 2 (Figures 2A,B). When comparing the virus group infused with vehicle to the virus group injected with letrozole a significant increase in pain was observed in week 1 and 2 (Figures 2A,B). A significant interaction between time and treatment was observed in the animals infused with letrozole in week 1 (*F*_(15,125)_ = 3.1, *p* < 0.001) and in week 2 (*F*_(15,125)_ = 1.9, *p* < 0.05).

In Experiment #3 the pain response in the control/vehicle, control/letrozol 5 mg/ml, VZV/vehicle and VZV/letrozole 5 mg/ml groups was similar to Experiment #2 (data not shown). Infusing the thalamus with 1 mg/ml letrozole resulted in no significant increase in the pain response vs. the VZV/vehicle group (Figures 2A,B). In addition, the pain response in the VZV group treated with 5 mg/ml of letrozole was significantly increased vs. the VZV group treated with 1 mg/ml of letrozole in both week 1 and 2 (Figures 2A,B).

### Gene Expression Analysis

**Figure 3:**
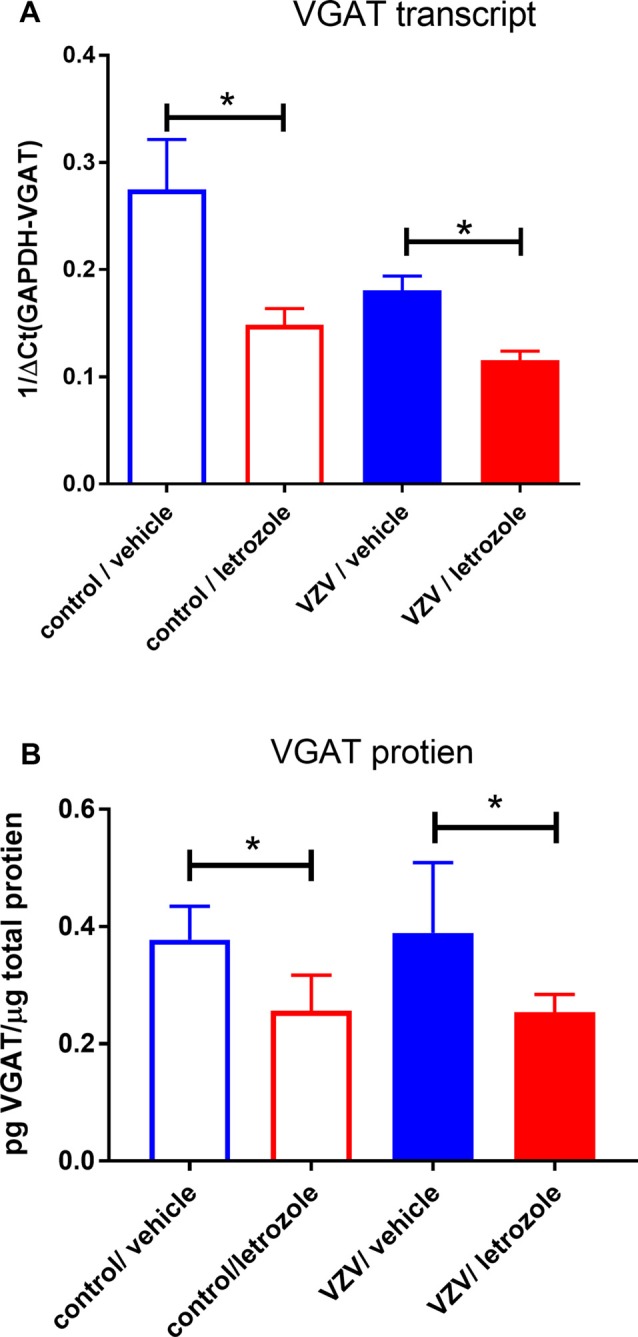
Vesicular GABA transporter (VGAT) expression in the thalamus was significantly reduced after infusing letrozole 5 mg/ml into the thalamus. Animals were sacrificed after the second week of place escape/avoidance paradigm (PEAP) testing. In panel **(A)**, real time polymerase chain reaction (RT-PCR) was completed after isolating thalamic plugs (four animals per group) and in panel **(B)** VGAT protein was quantitated after isolating thalamic plugs by enzyme-linked immunosorbent assay (ELISA; five animals per group). The asterisks indicate a significant difference of *p* < 0.05. Values are means and SEM.

Letrozole treatment decreased VGAT expression in the thalamus four-fold after VZV injection and six-fold in the control group. The change in expression was significant when analyzing the Ct values (Figure 3A). In these same animals no significant change in androgen receptor, aromatase CYP19a1, ER alpha (ERα), ER beta (ERβ) or G protein-coupled receptor 30 (GPR30) transcript was observed after letrozole treatment (data not shown). Protien content significantly decreased after letrozole treatment consistent with the transcript results (Figure 3B).

### Neuronal Activity in the Thalamic Nuclei

**Figure 4:**
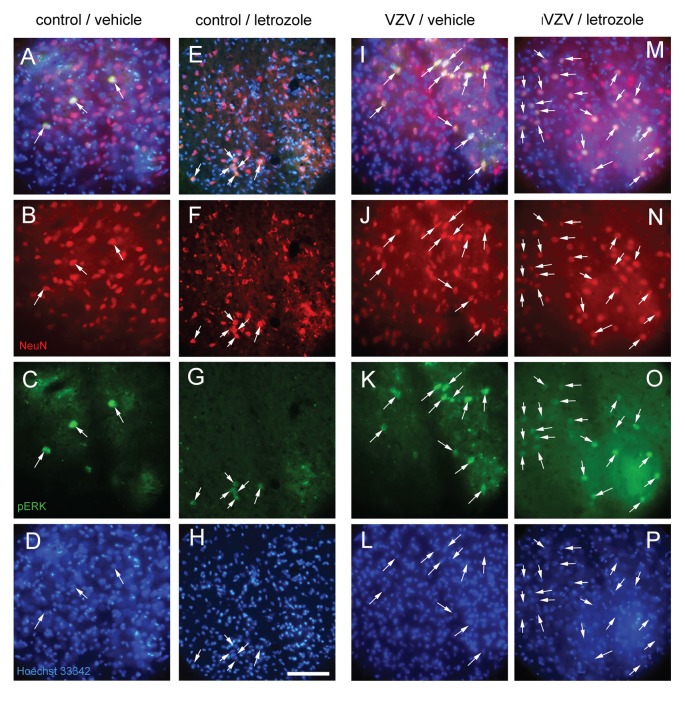
Neuronal nuclei (NeuN) and phosphorylated ERK (pERK) stained cells within the lateral thalamic region. NeuN stained cells (red, panels **B,F,J,N**, arrows) that co-localize with pERK (green, panels **C,G,K,O**, arrows) are shown as yellow cells (panels **A,E,I,M** in rats after whisker pad injection of VZV or control and then infusion (lateral thalamus) with letrozole (5 mg/ml) or vehicle. A representative animal injected with control and then infused with vehicle is shown in panels **(A–D)**. Panels **(E–H)** shows a representative image from an animal injected with control and infused with letrozole, panels **(I–L)** are from an animal injected with VZV and infused with vehicle and panels **(M–P)** are from a rat injected with VZV and infused with letrozole. Nuclei are stained with Hoechst 33342 in panels **(D,H,L,P)**. Bar = 100 micrometers.

**Figure 5:**
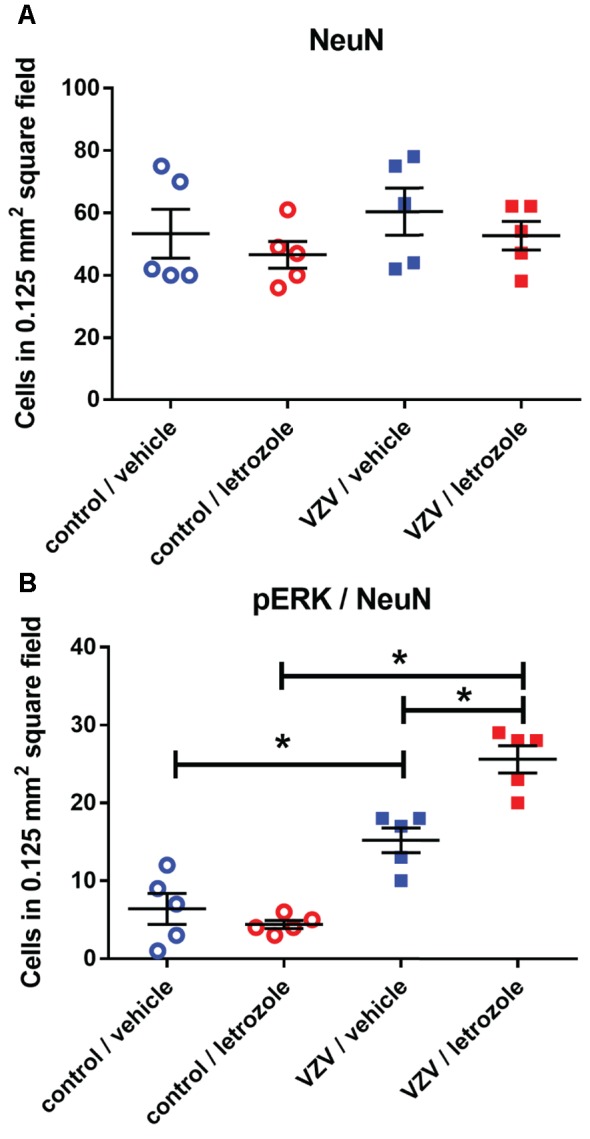
Cell counts within the lateral thalamic region for cells stained for NeuN **(A)** or NeuN and pERK **(B)** after injecting VZV or control into the whisker pad and then infusing letrozole (5 mg/ml) or vehicle into the lateral thalamus. The asterisks indicate a significant difference of *p* < 0.05. There were five animals in each treatment group. Values are means and SEM.

pERK can be a marker for pain-induced neuronal activation (Gao and Ji, 2009). NeuN stained cells were present in the thalamic area around the infusion site (Figures 4B,F,J,N). Cells in the same area also stained for pERK (Figures 4C,G,K,O). NeuN stained cells co-localized with pERK staining (Figures 4A,E,I,M). A blue nuclear stain of the sections in Figure 4 is shown in panels Figures 4D,H,L,P. Quantitation of the NeuN stained cells indicated no significant difference between the treatment groups (Figure 5A) but the number of NeuN/pERK stained cells increased significantly in the lateral thalamic region after VZV injection (Figure 5B). Treatment with letrozole significantly increased the number of NeuN/pERK stained cells in rats infected with VZV (Figure 5B).

### Neuronal Activity Within Rt GABA Cells

**Figure 6:**
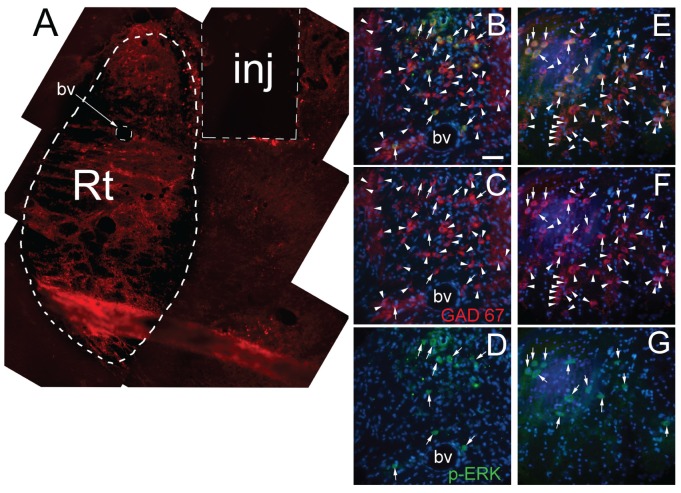
Gaba amino decarboxylase 67 (GAD 67) and pERK stained cells within the lateral thalamic region. Panel **(A)** shows a representative low magnification image of the ventral posteromedial (VPM), ventral posterolateral (VPL) and reticular thalamic nuclei (Rt) region from a VZV/letrozole (5 mg/ml) treated rat. The path of the injection needle (inj, dotted box) is within the VPM/VPL. GAD 67 stained cells (red) are localized within the Rt (dotted region). Panels **(B,C,D)** show a high magnification image of the Rt region from panel **(A)**. Note the blood vessel (bv) for orientation. Red GAD 67 stained cells in panels (**C,F**; indicated by arrows and arrowheads) and pERK stained cells in panels (**D,G**; green cells, arrows) co-localize (yellow cells) in panels (**B,E**; arrows). Nuclei were stained with Hoechst 33342 (blue). Bar = 50 micrometers.

**Figure 7:**
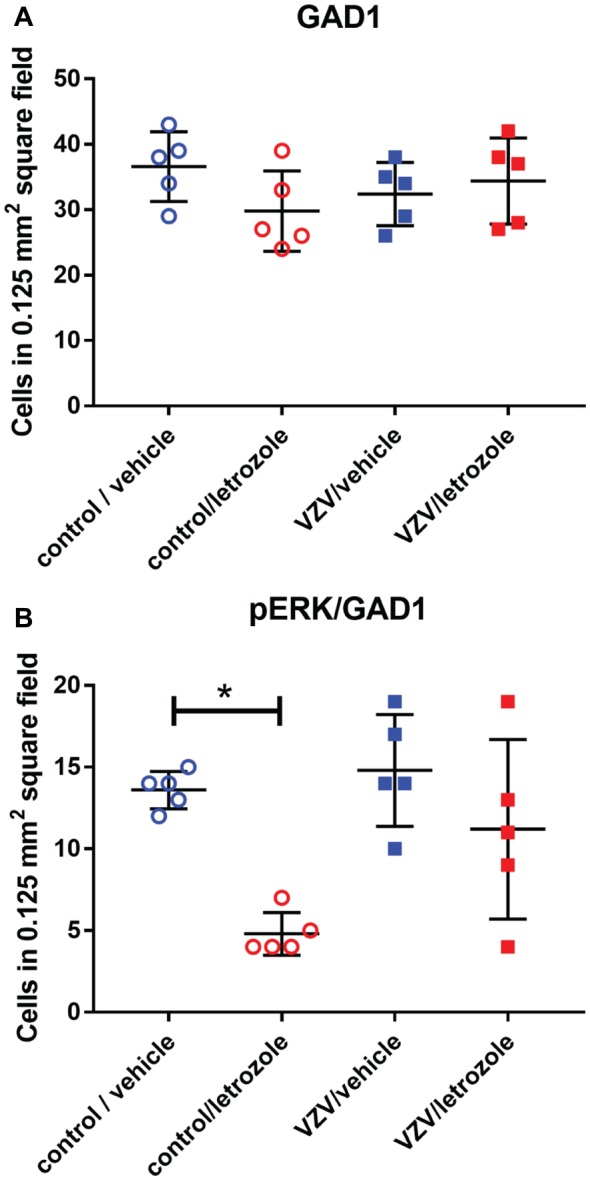
Cell counts within the lateral thalamic region for cells stained for GAD 67 only **(A)** or GAD 67 and pERK **(B)** after injecting VZV or control into the whisker pad and then infusing letrozole (5 mg/ml) or vehicle into the lateral thalamus. The asterisks indicate a significant difference of *p* < 0.05. There were five animals in each treatment group. Values are means and SEM.

GABA cells within the lateral thalamic region are primarily located in the Rt nucleus (Figure 6A). A few GAD 67 positive cells expressed pERK (Figures 6B–G). Counts of GAD 67 positive cells within the Rt indicated no change between treatment groups (Figure 6A). Interestingly, no change in pERK expression was observed in these cells (Figure 7) with exception of the control group, showing letrozole decreased the number of GAD 67/pERK positive cells (Figure 7B).

### Thalamic Infusion of ERα Agonist Attenuated the Pain Response

**Figure 8:**
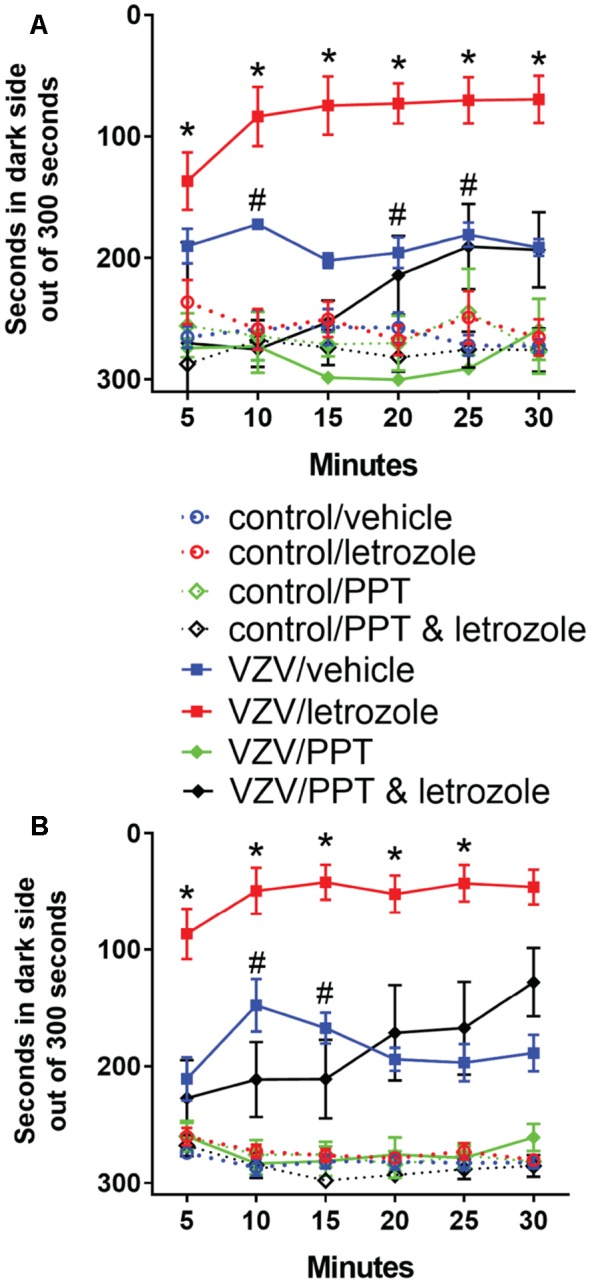
Local thalamic infusion of the estrogen receptor (ER) agonist 4, 4′, 4″-(4-propyl-[1*H*]-pyrazole-1,3,5-triyl)trisphenol (PPT) concomitant with letrozole (5 mg/ml) significantly reduced the VZV induced pain response vs. letrozole treatment alone. Note that the data includes data from Figure 2. Hashtag symbol represents a significant difference (*p* < 0.05) between the VZV/vehicle group and the VZV/PPT group. The asterisks indicate a significant difference (*p* < 0.05) between the VZV/letrozole (red solid square symbols) and VZV/PPT & letrozole group (solid black diamond symbols) *n* = 8 per group. Panel **(A)** is week 1 data and panel **(B)** is week 2 data Values are means and SEM.

Because blocking local, thalamic production of estradiol increased the pain response we asked if the estradiol might be working through the ER. To test this idea, ER agonist PPT was infused in the lateral thalamic region. PPT significantly reduced (*p* < 0.05) the pain response following VZV injection (compare the VZV/vehicle group to the VZV/PPT group, Figures 8A,B). PPT also significantly reduced (*p* < 0.05) the response in the letrozole treated animals (compare VZV/letrozole to VZV/PPT and letrozole, Figures 8A,B).

### Letrozole Increased pERK Staining After PPT Treatment

**Figure 9:**
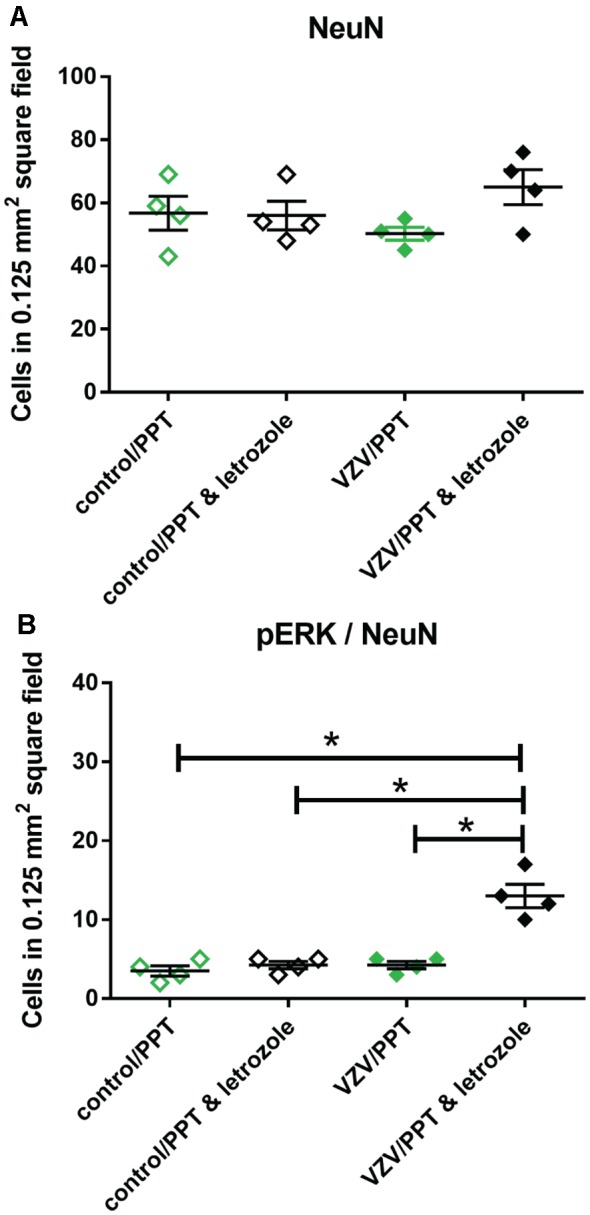
Cell counts within the lateral thalamic region for cells stained for NeuN **(A)** or NeuN and pERK **(B)** after injecting VZV or control into the whisker pad and then infusing letrozole (5 mg/ml) and/or PPT into the lateral thalamus. Asterisks indicate a significant difference between groups (*p* < 0.05). There were four animals counted in each treatment group. Values are means and SEM.

The number of NeuN stained cells in the lateral thalamic region was not affected by treatment (Figure 9A). Infusion of PPT and letrozole significantly increased (*p* < 0.05) the number of NeuN/pERK stained cells in the lateral thalamic region 2 weeks after VZV injection (Figure 9B). Lateral thalamic infusion of ER agonist PPT significantly decreased (*p* < 0.05) the number of pERK stained neurons in the letrozole treated rats injected with VZV (compare the VZV/letrozole group in Figure 5B to the VZV/PPT and letrozole group in Figure 9B).

## Discussion

Injecting the whisker pad with VZV results in pain and letrozole increases the VZV associated pain. The thalamus has an active role in orofacial pain responses (Sessle, 1986; de Leeuw et al., 2005; Yen and Lu, 2013). Lesions of the thalamus increase the pain response (Saadé et al., 1999). In humans, aromatase levels are high in the thalamus, pons, hypothalamus, amygdala and hippocampus (Sasano et al., 1998; Azcoitia et al., 2011). Letrozole is an inhibitor of aromatase, the enzyme that catalyzes the formation of estradiol from testosterone, and this enzyme is active within the thalamus (Biegon et al., 2010). Letrozole increased the number of pERK positive neurons within the thalamus of VZV injected rats. Importantly, the number GAD 67 positive cells labeling with pERK remained unchanged after letrozole treatment suggesting GABA neurons within the Rt were not more active. This increase in pERK positive neurons after letrozole treatment was likely representing increased activity within the excitatory neuronal population.

One mechanism by which estradiol could alter neuronal activity would be GABA. A previous report from our lab demonstrated that estradiol can increase expression of GABA related genes in the thalamus (Umorin et al., 2016). Previous studies from our lab also suggested thalamic expression of VGAT attenuates pain in the orofacial region (Kramer and Bellinger, 2009; Kramer et al., 2015). Because GABA attenuates the pain response and estradiol reduces pain we surmised that estradiol could reduce pain by increasing GABA expression (Umorin et al., 2016). Letrozole treatment decreased VGAT transcript and protein within the thalamus. This decrease in VGAT would reduce GABA signaling and increase excitatory neuronal activity. Increasing excitatory activity in the thalamus should increase the pain response (Osikowicz et al., 2013; Acher and Goudet, 2015). Increased pain after letrozole treatment was consistent with the idea that inhibiting aromatase decreased GABA inhibition of excitatory neurons in the thalamus enhancing the pain response.

Changes in VGAT expression resulting from estradiol have been reported in the hypothalamic region (Ottem et al., 2004; Noriega et al., 2010) and our lab has reported changes in VGAT expression within the thalamus throughout the estrous cycle (Umorin et al., 2016). Multiple potential estrogen response element sites are present in the VGAT promoter (Hudgens et al., 2009). Although ERα is not expressed at significant levels within the Rt, ERβ has been observed in the rostral region of the Rt (Lein et al., 2007). PPT is highly selective for ERα but the agonist could have stimulated ERβ receptors to effect VGAT expression. The increased pain response after letrozole treatment was reversed after PPT treatment a result that could be explained by PPT signaling ERβ to increase VGAT production.

Estradiol can attenuate pain through several nuclear or membrane receptors (Perez et al., 2003; Brailoiu et al., 2007; Hazell et al., 2009). Estrogen effects pain transmission via membrane bound ERs (Zhang et al., 2012). 17β-estradiol, the most potent form of estrogen, regulates gene expression of voltage gated sodium channels via ERα and ERβ, which may play a vital role in modulation of pain (Hu et al., 2012). Thus, estrogen can modulate pain acting through any of its receptors in a genomic or non-genomic mechanism (Craft, 2007; Deliu et al., 2012; Amandusson and Blomqvist, 2013). We measured expression of androgen and ERs. There was no change in expression of these receptors after letrozole administration. Moreover, aromatase expression did not change. These results suggest that letrozole does not affect expression of these genes and that the mechanism increasing VZV associated pain does not include changes in expression of these genes.

Letrozole treatment often results in excacerbating the pain response after insult (Ghorbanpoor et al., 2014; Robarge et al., 2016). Consistent with these reports our results showed an increased VZV pain response after letrozole treatment. Humans report pain after taking aromatase inhibitors but we did not observe an increased response in non-VZV animals taking letrozole (Henry et al., 2008, 2012). Inhibition of aromatase increases mechanical hyperalgesia in animals but not thermal hyperalgesia suggesting aromatase effects are varied depending on the response being measured (Robarge et al., 2016). Thus, it is possible we did not see an increased pain response in the control/letrozole groups because letrozole by itself (i.e., without VZV pain) results in changes to mechanical hyperalgisia but not motivational/affective aspect of pain as measured in this study.

Because letrozole treatment increased the VZV induced pain response, we suggest estradiol in the thalamus inhibits orofacial pain. Previous studies have suggested that estradiol attenuates orofacial pain consistent with these results (Fischer et al., 2008; Kramer and Bellinger, 2009, 2013; Tashiro et al., 2012; Stinson et al., 2017) but this is the first study to demonstrate a role for estradiol in the lateral thalamic region attenuating VZV pain. Moreover, it was apparent that much of the estradiol was produced locally because local administration of an aromatase inhibitor resulted in a heightened pain response. This is an interesting result because increased systemic estradiol has also been associated with an attenuated pain response (Stinson et al., 2017). Thus, systemic estradiol and estradiol produced from testosterone in the thalamus both alter the pain response, but systemic estradiol possibly works through a different mechanism.

The Rt projects to regions such as the parafascicular and intralaminar nuclei (Clemente-Perez et al., 2017). Evidence suggests the parafascicular nucleus is important in motivational and effective pain (Weigel and Krauss, 2004) thus, altered VGAT expression in the Rt could alter the VZV pain response measured in this study. Alternatively, letrozole could have spread to the zona incerta (ZI) after infusion into the thalamus. The ZI contains ERs and aromatase (Jakab et al., 1993; Shughrue et al., 1997). The mediodorsal thalamus (MD) receives dense GABAergic inputs from the ZI (Bartho et al., 2002; Erickson et al., 2004) and is heavily connected to cortical areas involved in processing the affective aspects of pain (Cornwall and Phillipson, 1988; Groenewegen, 1988). Thus, changes in estradiol signaling within the ZI could alter the VZV pain response through modulation of GABAergic cells.

From the PEAP graphs, some difference was seen between the results from the systemic and cannulated groups. This difference may result from local administration of letrozole providing a higher concentration to the regulatory site vs. a systemic injection. To verify that a reduction in the letrozole concentration would reduce the behavioral response, we performed another experiment injecting the brain with a five-fold lower dose or 1 mg/ml; (2 mg/kg ÷ 5-fold lower) = 0.4 mg/kg dose. Note: a 0.5 mg/kg dose was not effective in blocking estrogen signaling in a prior study (Liu et al., 2015). Our results were consistent with the Liu study, such that, infusing a concentration of 1 mg/kg (0.4 mg/kg dose) did not elicit a significant behavioral response.

In summary, inhibiting aromatase activity increased pERK in the non-GABAergic neuronal population and increased the pain response in male rats. VGAT expression in the thalamus was reduced after letrozole treatment. PPT treatment reversed letrozole effects by decreasing pain and reducing the number of pERK positive neurons consistent with the idea that locally produced estrogen binds the ER, upregulating VGAT resulting in neuronal inhibition and attenuation of the pain response.

## Author Contributions

All authors contributed to the planning and devised the experiments.

## Conflict of Interest Statement

The authors declare that the research was conducted in the absence of any commercial or financial relationships that could be construed as a potential conflict of interest.

## Abbreviations

DMSO: dimethyl sulfoxide
ELISA: Enzyme-linked immunosorbent assay
ERα: estrogen receptor alpha
ERβ: estrogen receptor beta
GABA: γ-aminobutyric acid
GAD 67: GABA amino decarboxylase 67
GAD 65: GABA amino decarboxylase 65
GAPDH: Glyceraldehyde-3-Phosphate Dehydrogenase
GPR30: G protein-coupled receptor 30
NeuN: neuronal nuclei
PBS: phosphate buffered saline
PEAP: Place Escape/Avoidance Paradigm
pERK: phosphorylated ERK
Po: posterior thalamic nucleus
PPT: ERα agonist 4, 4′,4″-(4-Propyl-[1*H*]-pyrazole-1,3,5-triyl)*tris*phenol
Rt: reticular thalamic nuclei
VGAT: vesicular GABA transporter
VPL: ventral posterolateral thalamic nuclei
VPM: ventral posteromedial
VZV: varicella zoster virus
ZI: zona incerta.
