# Corrigendum: Aromatase Derived Estradiol Within the Thalamus Modulates Pain Induced by Varicella Zoster Virus

**DOI:** 10.3389/fnint.2018.00066

**Published:** 2019-01-31

**Authors:** Phillip R. Kramer, Mahesh Rao, Crystal Stinson, Larry L. Bellinger, Paul R. Kinchington, Michael B. Yee

**Affiliations:** ^1^Department of Biomedical Science, Texas A&M University College of Dentistry, Dallas, TX, United States; ^2^Department of Ophthalmology and of Molecular Microbiology and Genetics, Eye and Ear Foundation, School of Medicine, University of Pittsburgh, Pittsburgh, PA, United States

**Keywords:** orofacial, herpes zoster, neuralgia, shingles, pain, estrogen, aromatase, thalamus

In the original article, there was a mistake in Figure 3 as published. The value of “mg” should have been “μg.” The corrected Figure 3 appears below. A correction has also been made to the Material and Methods, Enzyme-linked immunosorbent assay (ELISA).

**Figure 3:**
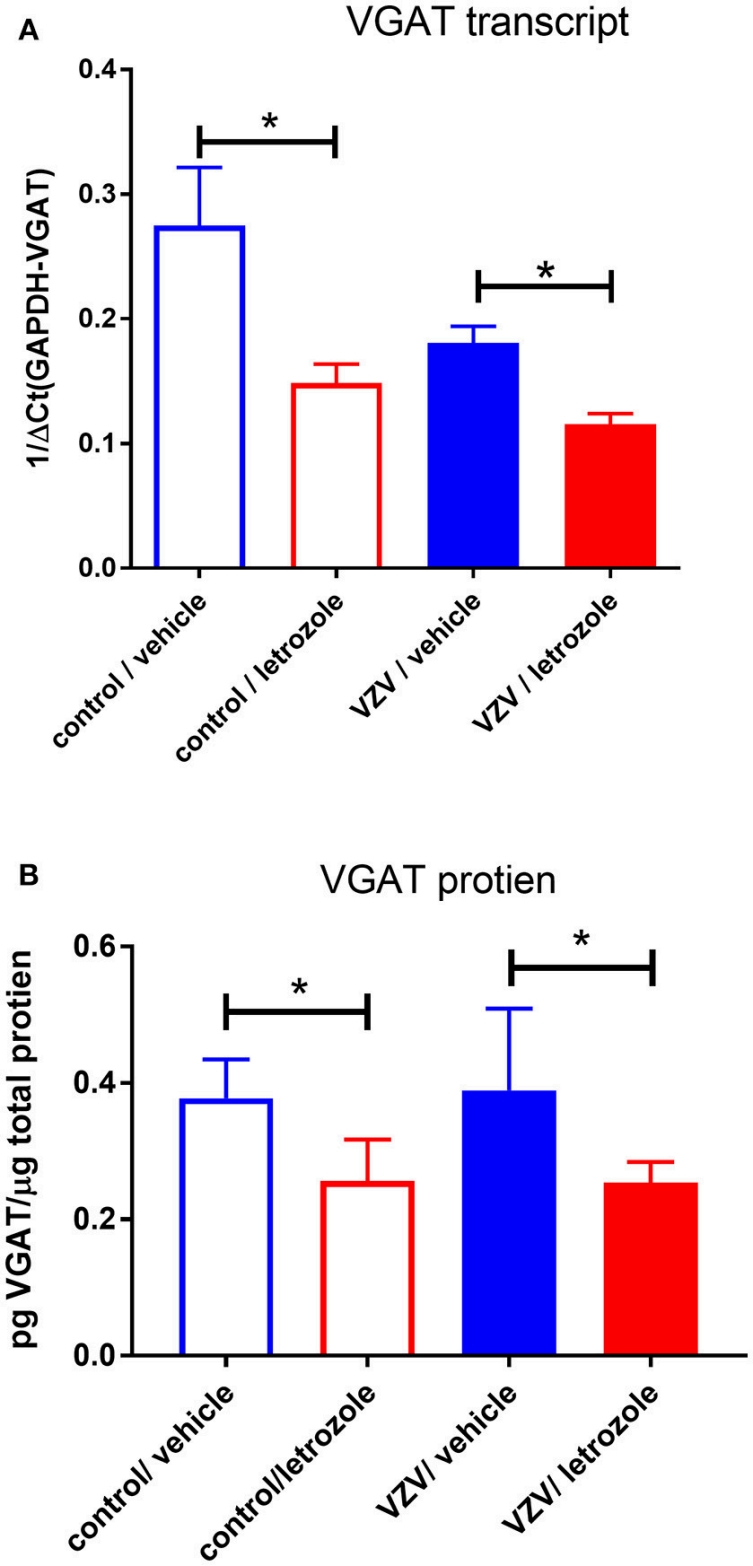
Vesicular GABA transporter (VGAT) expression in the thalamus was significantly reduced after infusing letrozole 5 mg/ml into the thalamus. Animals were sacrificed after the second week of place escape/avoidance paradigm (PEAP) testing. In panel **(A)**, real time polymerase chain reaction (RT-PCR) was completed after isolating thalamic plugs (four animals per group) and in panel **(B)** VGAT protein was quantitated after isolating thalamic plugs by enzyme-linked immunosorbent assay (ELISA; five animals per group). The asterisks indicate a significant difference of *p* < 0.05. Values are means and SEM.

“Fresh thalamic tissue punches from all five animals in experiment #3 (Table 1) was stored in liquid nitrogen until analysis. Tissue was placed in 250 μl of T-Per tissue protein extraction reagent containing Halt Protease Inhibitor and ground (Thermo Scientific, Rockford, IL, USA). Ground samples were frozen and thawed, followed by centrifugation and decanting of the supernatant. Quantitation of VGAT in the supernatant was completed on duplicate 100 μl samples of supernatant using an SLC32A1 (VGAT) ELISA following the manufacturer's directions (Cusabio, catalog #CSB-EL021578MO). Total protein was determined in each sample using a BCA protein assay (Thermo Scientific, Waltham, WA, USA). Values represent the pg of VGAT per μg of total protein.”

Additionally, the middle initial was missing in the first author's name. This has been corrected from “Philip Kramer” to “Philip R. Kramer.”

The authors apologize for this error and state that this does not change the scientific conclusions of the article in any way. The original article has been updated.

## Conflict of Interest Statement

The authors declare that the research was conducted in the absence of any commercial or financial relationships that could be construed as a potential conflict of interest.

